# IL-15 superagonist/IL-15RαSushi-Fc fusion complex (IL-15SA/IL-15RαSu-Fc; ALT-803) markedly enhances specific subpopulations of NK and memory CD8+ T cells, and mediates potent anti-tumor activity against murine breast and colon carcinomas

**DOI:** 10.18632/oncotarget.7470

**Published:** 2016-02-18

**Authors:** Peter S. Kim, Anna R. Kwilas, Wenxin Xu, Sarah Alter, Emily K. Jeng, Hing C. Wong, Jeffrey Schlom, James W. Hodge

**Affiliations:** ^1^ Laboratory of Tumor Immunology and Biology, Center for Cancer Research, National Cancer Institute, National Institutes of Health, Bethesda, MD, USA; ^2^ Altor BioScience Corporation, Miramar, FL, USA

**Keywords:** IL-15, IL-15 superagonist, NK cells, immunotherapy

## Abstract

Interleukin (IL)-15-N72D superagonist-complexed with IL-15RαSushi-Fc fusion protein (IL-15SA/IL-15RαSu-Fc; ALT-803) has been reported to exhibit significant anti-tumor activity in murine myeloma, rat bladder cancer, and murine glioblastoma models. In this study, we examined the immunomodulatory and anti-tumor effects of IL-15SA/IL-15RαSu-Fc in tumor-free and highly metastatic tumor-bearing mice. Here, IL-15SA/IL-15RαSu-Fc significantly expanded natural killer (NK) and CD8^+^ T cells. In examining NK cell subsets, the greatest significant increase was in highly cytotoxic and migrating (CD11b^+^, CD27^hi^; high effector) NK cells, leading to enhanced function on a per-cell basis. CD8^+^ T cell subset analysis determined that IL-15SA/IL-15RαSu-Fc significantly increased IL-15 responding memory (CD122^+^, CD44^+^) CD8^+^ T cells, in particular those having the innate (NKG2D^+^, PD1^−^) phenotype. In 4T1 breast tumor–bearing mice, IL-15SA/IL-15RαSu-Fc induced significant anti-tumor activity against spontaneous pulmonary metastases, depending on CD8^+^ T and NK cells, and resulting in prolonged survival. Similar anti-tumor activity was seen in the experimental pulmonary metastasis model of CT26 colon carcinoma cells, particularly when IL-15SA/IL-15RαSu-Fc was combined with a cocktail of checkpoint inhibitors, anti-CTLA-4 and anti-PD-L1. Altogether, these studies showed for the first time that IL-15SA/IL-15RαSu-Fc (1) promoted the development of high effector NK cells and CD8^+^ T cell responders of the innate phenotype, (2) enhanced function of NK cells, and (3) played a vital role in reducing tumor metastasis and ultimately survival, especially in combination with checkpoint inhibitors.

## INTRODUCTION

IL-15 is a pleiotropic cytokine that has been shown to induce and regulate a wide range of immune functions [1, 2]. Specifically, IL-15 is critical for lymphoid development and peripheral maintenance of innate immune cells and immunological memory of T cells, in particular natural killer (NK) and CD8^+^ T cell populations [1, 2]. However, while IL-15 does not promote the maintenance of CD4^+^ CD25^+^ FOXP3^+^ regulatory T cells (Tregs), IL-2 has been demonstrated to induce their development [3–5]. Furthermore, IL-15 has been shown to protect effector T cells from IL-2–mediated activation-induced cell death (AICD) [6, 7]. For these reasons, IL-15 has long been speculated to have high therapeutic potential for long-term anti-tumor immunity. The cytokine's immune effect in cancer patients has been recently examined. In a first-in-human clinical trial of recombinant human (rh)IL-15, Conlon et al. found that rhIL-15 administration induced a 10-fold expansion of NK cells and significantly increased the proliferation of γδT cells and CD8^+^ T cells [8].

Despite its promising anti-tumor immune capacity, IL-15 has been shown to exhibit a short half-life and high doses were required to achieve biological responses *in vivo* [9, 10], hence resulting in clinical toxicities and limited anti-tumor responses in patients [8]. To increase the therapeutic effectiveness and facilitate the use of IL-15 in the immunotherapy of cancer and chronic infection, an IL-15 N72D superagonist/IL-15RαSushi-Fc fusion complex (IL-15SA/IL-15RαSu-Fc; ALT-803) has been developed to address some of the limitations of IL-15–based therapeutics. First, in the IL-15 N72D superagonist (IL-15SA), the asparagine 72 was replaced with the aspartic acid residue, providing improved affinity for CD122-expressing immune cells and promoting stronger cytoplasmic signals for activation and proliferation of NK and CD8^+^ T cells at lower dosages [11]. Furthermore, it has been previously shown that the biological activity of IL-15 increased when IL-15 was pre-complexed with IL-15Rα [12, 13]. Simulating trans-presentation between dendritic cells/macrophages and effector cells, the sushi domain of IL-15Rα, fused to the Fc portion of human IgG1 [11], has been engineered to incorporate the trans-presentation mechanism, consequently increasing the half-life and *in vivo* biological activity of the IL-15-SA [11, 14]. Overall, when compared with native IL-15, the IL-15SA/IL-15RαSu-Fc fusion complex has been shown to exhibit a longer serum half-life and retention in lymphoid organs and increased *in vivo* biological activity by 5–25-fold [11, 14, 15].

Due to its potent immunostimulatory capability, the IL-15SA/IL-15RαSu-Fc fusion complex has been shown to be efficacious in several experimental animal models of cancer, namely murine multiple myeloma [16], rat bladder cancer [17], and murine glioblastoma [18], and currently is being tested against human hematological and solid cancers in multiple clinical trials (ClinicalTrials.gov). Here, we evaluated for the first time, (1) the immunomodulatory effect of IL-15SA/IL-15RαSu-Fc on the subpopulations of NK cells (and memory CD8^+^ T cells) and (2) its anti-tumor activity against pulmonary metastases in the 4T1 breast and CT26 colon carcinoma models, with the aim of providing a rationale for the utilization of IL-15SA/IL-15RαSu-Fc, especially in combination with checkpoint inhibitors, in the immunotherapy of highly metastatic cancers.

## RESULTS

### IL-15SA/IL-15RαSu-Fc induced marked elevations of TH_1_ and TH_2_ cytokines

**Figure 1:**
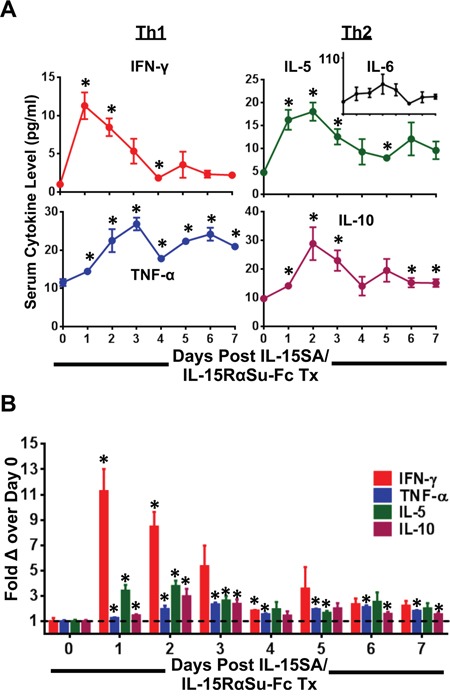
IL-15SA/IL-15RαSu-Fc markedly induces TH_1_ and TH_2_ cytokines A multiplex cytokine analysis is shown, measuring **A.** serum cytokine concentrations of IFN-γ, TNF-α, IL-5 and IL-10 as well as **B.** their fold changes over a 7-day period post treatment in female Balb/c mice (n=3/group). Serum IL-6 level is shown as an inset in (A). *p < 0.05, statistical significance.

Due to the pleiotropic nature of IL-15 in regulating various immune responses, we first sought to examine the extent to which IL-15SA/IL15-RαSu-Fc promoted the production of Th1 and Th2 cytokines over a 7-day period. Mice administered with IL-15SA/IL15RαSu-Fc exhibited a transient increase in the serum concentration levels of IFN-γ, TNF-α, IL-5, and IL-10 (Figure 1A). Serum IFN-γ level, in particular, peaked on day 1 (*p =* 0.004), followed by IL-5 and IL-10 on day 2 (*p =* 0.005 and *p =* 0.030, respectively), then TNF-α on day 3 (*p =* 0.001) (Figure 1A). There was no significant change observed in serum IL-6 level (Figure 1A; inset). The greatest fold change was observed for IFN-γ, whose fold increase was as high as ∼11-fold (*p =* 0.004) on day 1, whereas the other cytokines did not increase beyond 5-fold during the 7-day period (Figure 1B). The duration of elevated serum cytokine level was the greatest for TNF-α, maintaining significantly above the baseline on day 7 (*p =* 0.001), and the shortest for IFN-γ, lasting up to day 4 (*p =* 0.028) (Figure 1A). Even though administration of IL-15SA/IL-15RαSu-Fc to mice rapidly increased inflammatory cytokines at the dose described, no observable toxicities were seen in mice throughout the 7-day period.

### IL-15SA/IL-15RαSu-Fc promoted the expansion of NK, T, B cell and granulocytic populations in the spleen

**Figure 2:**
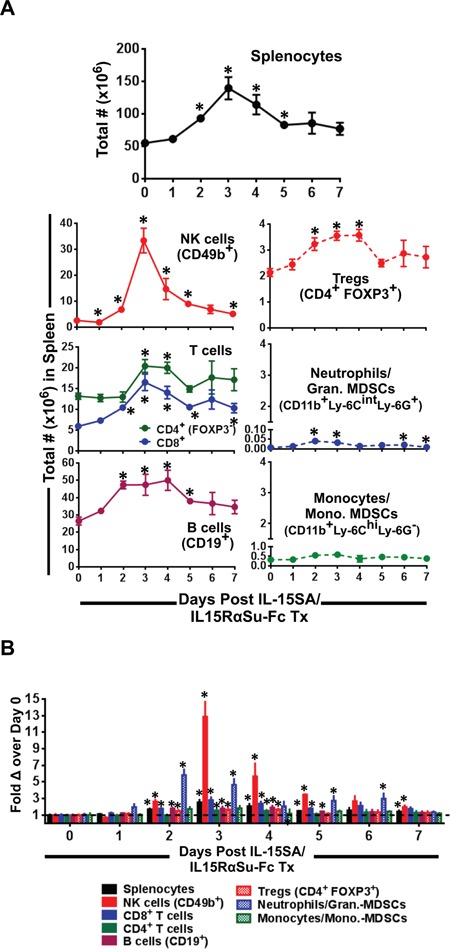
IL-15SA/IL-15RαSu-Fc significantly induces the expansion of splenic NK, T, B cell and granulocytic populations **A.** The total numbers of splenocytes, NK (CD49b^+^) cells, T (CD8^+^ and CD4^+^) cells, B (CD19^+^) cells, Tregs (CD4^+^ FOXP3^+^), neutrophils/granulocytic MDSCs (CD11b^+^ Ly-6C^int^ Ly-6G^+^) and monocytes/monocytic MDSCs (CD11b^+^ Ly-6C^hi^, Ly-6G^−^) as well as **B.** their fold changes are shown over a 7-day period post treatment in female Balb/c mice (n=3/group). *p < 0.05, statistical significance. This experiment was repeated at least 2 times with similar results.

Next, we examined the effect of IL-15SA/IL15RαSu-Fc on major immune populations in the spleen. Administration of IL-15SA/IL-15RαSu-Fc to mice induced the greatest effect on NK cells, whose increase in the total number was highest on day 3 (*p =* 0.003) and lasted markedly above the baseline up to day 5 (*p* < 0.001). T and B cells were similarly affected, as the total numbers of CD8^+^ and conventional (conv.) CD4^+^ T cells increased, peaking on day 3 (CD8^+^: *p =* 0.007 ; conv.CD4^+^: *p =* 0.013), whereas B cells and regulatory CD4^+^ T cells (Tregs) peaked on day 2 (B cells: *p =* 0.003; Tregs: *p =* 0.018) then plateaued until day 4 (B cells: *p =* 0.020; Tregs: *p =* 0.006) (Figure 2A). Among these lymphocytes, the highest fold change was observed in NK cells (∼13 fold on day 3; *p =* 0.003) followed by CD8^+^ T cells (∼3 fold on day 3; p = 0.007) then CD4^+^ (conv. and Tregs) T and B cells. We also examined the effect of IL-15SA/IL-15RαSu-Fc on granulocytic and monocytic populations. The total number of CD11b^+^ Ly-6C^int^ Ly-6G^+^ cells, identified as either neutrophils or granulocytic myeloid derived suppressor cells (MDSCs), increased as high as 6-fold on day 2 (*p =* 0.002), whereas CD11b^+^ Ly-6C^hi^ Ly-6G^−^ cells, characterized either as monocytes or monocytic-MDSCs, appeared to be unaffected by IL-15SA/IL-15RαSu-Fc. IL-15SA/IL-15RαSu-Fc similarly enhanced the expansion of NK cells in PBMCs in comparison with those in the spleens (Supplemental Figure 1). Taken together these data are consistent with the overall immunomodulatory property of IL-15, which activates both the innate and adaptive arms of the immune system [2, 19].

### IL-15SA/IL-15RαSu-Fc most affected IL-15-responding memory T cells, specifically of the innate phenotype in the CD8^+^ T cell compartment

**Figure 3:**
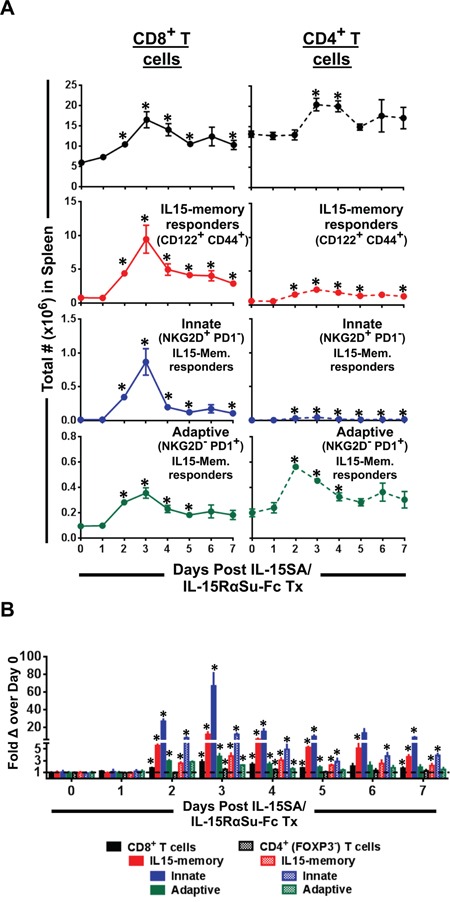
IL-15SA/IL-15RαSu-Fc increases IL-15 memory responders in splenic CD8^+^ T cell population, in particular those with the innate phenotype **A.** The total numbers of IL-15 memory responders (CD122^+^ CD44^+^) plus their innate (NKG2D^+^ PD1^−^) and adaptive (NKG2D^−^ PD1^+^) subsets in splenic CD8^+^ and CD4^+^ T cell populations as well as **B.** their fold changes are shown over a 7-day period post treatment in female Balb/c mice (n=3/group). *p < 0.05, statistical significance. This experiment was repeated at least 2 times with similar results.

We observed that even though the total numbers of both CD8^+^ and CD4^+^ T cells increased, partly as a result of overall increase in the total number of splenocytes, the fold increase was greater in CD8^+^ than in CD4^+^ T cells or splenocytes (Figure 2B). We therefore investigated which splenic T cell subsets, in particular in the CD8^+^ compartment, responded to IL-15SA/IL-15RαSu-Fc treatment. More specifically, we sought to examine the following phenotypically defined T cell subsets: (1) IL-15–responding memory T cells identified by the IL-15 receptor β-chain, CD122, and the antigen-experienced memory marker, CD44, (2) innate IL-15-responding memory T cells characterized by expression of the NK-activating receptor, NKG2D, on CD122^+^ CD44^+^ T cells, and (3) adaptive IL-15-responding memory T cells identified by an immunoinhibitory receptor, PD-1, which upregulates upon T cell receptor (TCR) ligation on CD122^+^ CD44^+^ T cells. Within the CD8^+^ T cell population, IL-15SA/IL-15RαSu-Fc significantly increased IL-15 memory responders (CD122^+^ CD44^+^; up to ∼15 fold on day 3; *p =* 0.014; Figure 3B) whose immune response profile was very similar to that of NK cells (Figures 2 and 3). Within IL-15 memory CD8^+^ T cell responders, those of the innate phenotype (NKG2D^+^ PD1^−^) increased dramatically as high as ∼70 fold on day 3 (*p =* 0.012) (Figure 3B), also having response kinetics similar to that seen in NK cells. However, IL-15SA/IL-15RαSu-Fc did not have a similar magnitude of effect on these subsets in the CD4^+^ T cell compartment (Figure 3A). Finally, in both CD8^+^ and CD4^+^ T cell compartments, IL-15SA/IL-15RαSu-Fc had an effect on IL-15 memory responders with the adaptive phenotype (NKG2D^−^ PD1^+^), but not to the same degree of magnitude as those with the innate phenotype (Figure 3B). These data are in line with IL-15SA/IL-15RαSu-Fc's capacity to convert antigen-experienced memory CD8^+^ T cells into non-antigen-specific innate effector cells as demonstrated by Xu et al. [16].

### IL-15-SA/IL-15RαSu-Fc promoted the development of “high effector” (CD11b^+^ CD27^hi^) NK cells

**Figure 4:**
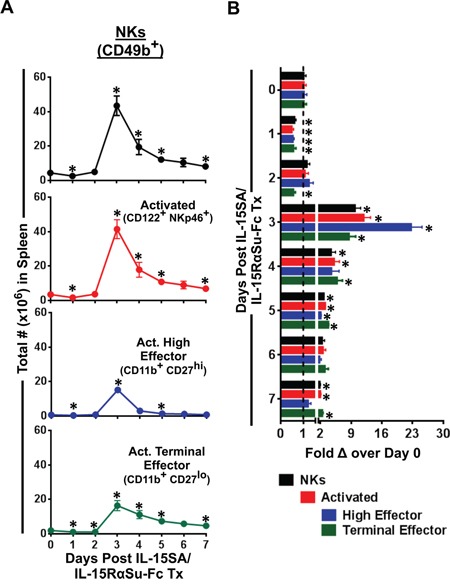
IL-15SA/IL-15RαSu-Fc enhances the development of “high effector” NK cells **A.** The total numbers of NK (CD49b^+^) cells, activated (CD122^+^ NKp46^+^) NK cells, activated “high effector” (CD11b^+^ CD27^hi^) and “terminal effector” (CD11b^+^ CD27^lo^) NK cells over a 7-day period post treatment in female Balb/c mice (n=3/group) as well as **B.** their fold changes are shown. *p < 0.05, statistical significance. This experiment was repeated at least 2 times with similar results.

Because IL-15SA/IL-15RαSu-Fc induced a potent expansion of NK cells in the spleen (Figure 2), we next investigated which splenic NK subset(s) most responded to IL-15SA/IL-15RαSu-Fc treatment. We found that unlike CD8^+^ T cells, nearly all NK cells throughout the 7-day period of IL-15SA/IL-15RαSu-Fc–mediated immune response had an activated phenotype (CD122^+^ NKp46^+^), leading us to further phenotype the NK population using CD11b and CD27 surface markers [20]. It has been reported that CD11b^+^ CD27^hi^ NK cells were resistant to self–major histocompatibility complex (MHC) class I–induced tolerance and exhibited high cytotoxicity, cytokine production and migratory capacity [20–23], hence termed “high effector” NK cells. On the other hand, CD11b^+^ CD27^lo^ or “terminal effector” NKs, albeit having high cytotolytic activity, produced low levels of cytokines, were less migratory, and highly expressed the terminal differentiation marker KLRG-1 [20–23]. The results showed that IL-15SA/IL-15RαSu-Fc significantly increased the total numbers of both high (CD11b^+^ CD27^hi^) and terminal (CD11b^+^ CD27^lo^) effector NK cells, peaking on day 3 (high effectors: *p =* 0.001; terminal effectors: *p =* 0.008) (Figure 4A), and the highest fold increase was observed in high effector NK cells (up to ∼20 fold on day 3; p = 0.001; Figure 4B). The capacity of IL-15SA/IL-15RαSu-Fc to generate high effector NK cells suggests that per-cell NK function may be increased as a result of the cytokine complex treatment.

### IL-15SA/IL-15RαSu-Fc increased total NK cells and function on a per-cell basis

**Figure 5:**
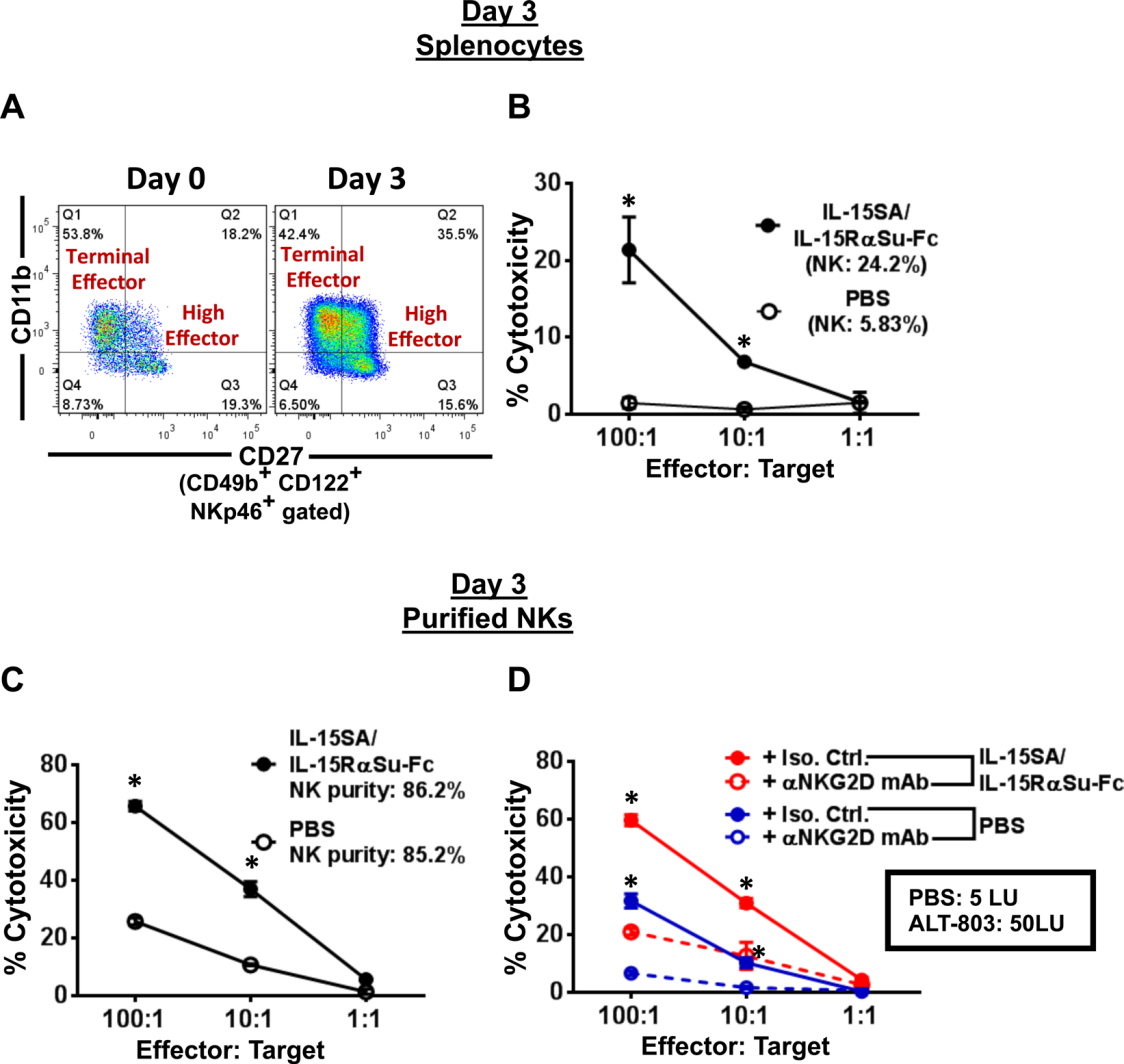
IL-15SA/IL-15RαSu-Fc increases the total-cell as well as per-cell function of NK cells, dependent on NKG2D Female Balb/c mice (n = 5–10/group) were given a single injection of PBS or IL-15-SA/IL-15RαSu-Fc, and the spleens were harvested on day 3 post-treatment. **A.** A representative flow analysis of splenic NK cell subsets from PBS and IL-15-SA/IL-15RαSu-Fc–treated mice is shown. **B.** Bulk splenocytes were used as effectors and co-cultured with YAC-1 target cells at 100:1, 10:1, and 1:1 effector-to-target ratios. ^111^In radioactivity was measured to determine cytotoxic function. **C.** Purified splenic NK cells were used as effectors in the cytotoxicity assay described in (B). **D.** Anti-mouse NKG2D mAb or its isotype control were added in the cytotoxicity assay described in (C); NK activity in lytic units (LU). All experiments were performed twice with similar results. Error bars represent SE of mean of quadruplicate measurements. *p < 0.05, statistical significance. This experiment was repeated 2 times with similar results.

Because IL-15SA/IL-15RαSu-Fc expanded high effector NK cells, we next investigated the effect of IL-15SA/IL-15RαSu-Fc on total and per-cell function of NK cells. On day 3, the proportion of high effector NK cells increased (∼2-fold on day 3), resulting in decreased proportions of other NK subsets (Figure 5A). When we examined the total NK activity using day 3 splenocytes as effectors and YAC-1 cells as targets, IL-15SA/IL-15RαSu-Fc dramatically increased total NK-mediated cytotoxicity (E:T/100:1, *p =* 0.004) (Figure 5B), which was consistent with the increased percentage of splenic NK cells from the IL-15SA/IL-15RαSu-Fc treatment group (∼4-fold on day 3; Figure 4A). We then purified NK cells from the spleens (purity: ∼85%) on day 3 to determine the effect of IL-15SA/IL-15RαSu-Fc on NK cells on a per-cell basis (Figure 5C). The results indicated that IL-15SA/IL-15RαSu-Fc significantly enhanced the per-cell function of NK cells (E:T/100:1, p< 0.001), mediated in large part by the presence of NKG2D, as the per-cell NK-mediated lytic activity (IL-15SA/IL-15RαSu-Fc: 50 LU vs. Isotype Ctrl: 5 LU) decreased when a blocking NKG2D antibody was used (Figure 5D). These studies indicate that IL-15SA/IL-15RαSu-Fc has the potential to induce a potent NK-mediated anti-tumor response due to its ability to enhance not only total NK function but also per-cell NK function through the generation of high effector NKs.

**Figure 6:**
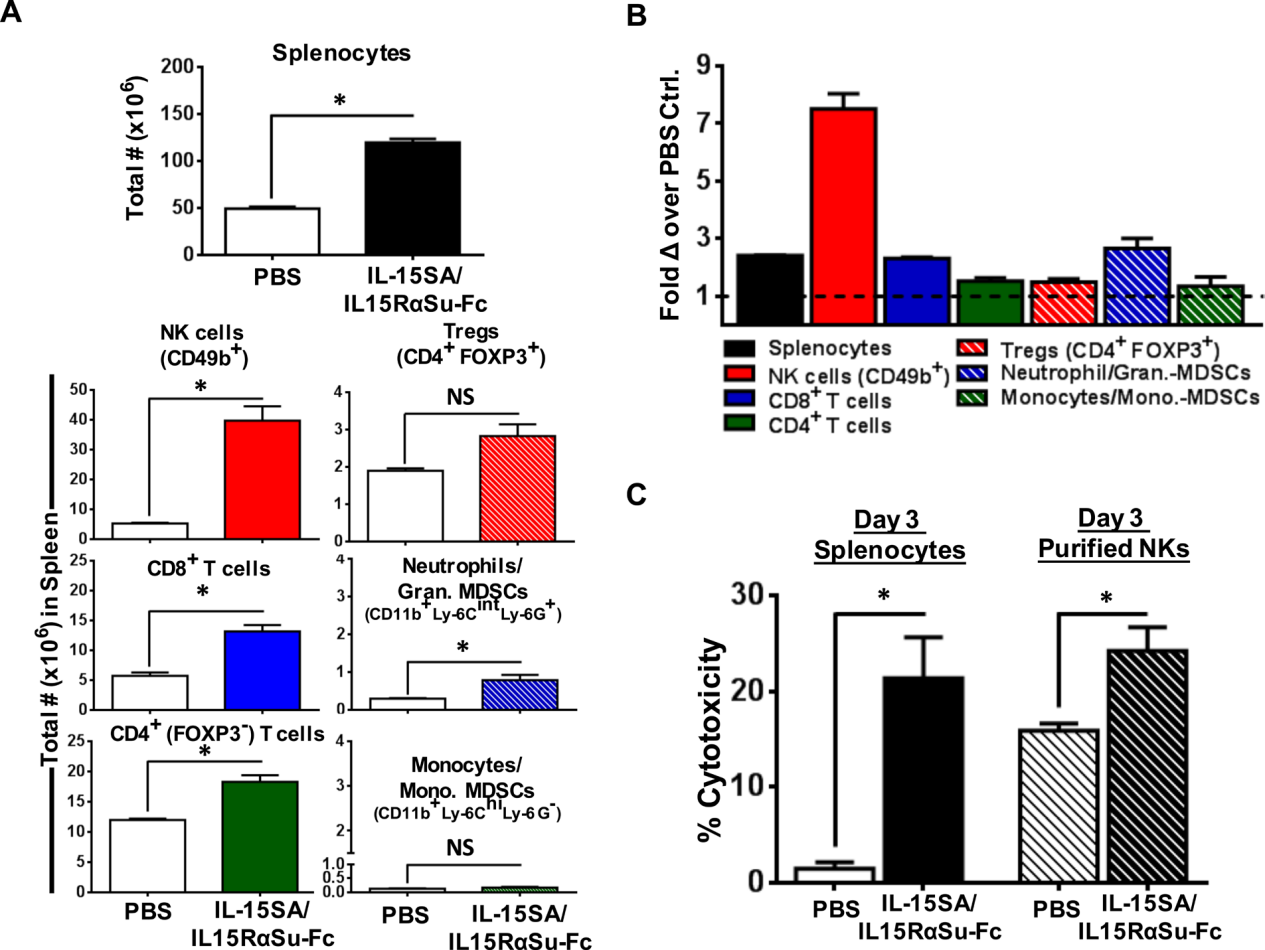
IL-15SA/IL-15RαSu-Fc significantly promotes the expansion of splenic lymphocyte and granulocyte populations in 4T1 tumor-bearing mice and increases *ex vivo* NK cytotoxicity against 4T1 tumor cells on total and per cell basis **A.** Female Balb/c mice (n=3/group) received 4T1 mammary tumor cells, then IL-15SA/IL-15RαSu-Fc or PBS on day 7 post–tumor implantation. On day 10, the spleens were harvested for immune analysis. The total numbers of splenocytes, NK (CD49b^+^) cells, T (CD8^+^ and CD4^+^) cells, Tregs (CD4^+^ FOXP3^+^), neutrophils/granulocytic MDSCs (CD11b^+^ Ly-6C^int^ Ly-6G^−^) and monocytes/monocytic MDSCs (CD11b^+^ Ly-6C^hi^, Ly-6G^+^) as well as **B.** their fold changes are shown. **C.** Female Balb/c mice (n = 5–10/group) were given a single injection of IL-15-SA/IL-15RαSu-Fc or PBS, and the spleens were harvested on day 3 post-treatment. Bulk splenocytes or purified NK cells were used as effectors and co-cultured with 4T1 tumor cells at a 100:1 effector-to-target ratio. ^111^In radioactivity was measured to determine cytotoxic function.

We next conducted studies in tumor-bearing mice to determine if treatment with IL-15SA/IL-15RαSu-Fc would impact immune cell populations to a similar degree as observed in non-tumor-bearing mice. We utilized the spontaneous metastasis model of 4T1 breast carcinoma. We injected 1 μg of IL-15SA/IL-15RαSu-Fc on day 7 post–tumor implantation when the primary tumor size ranged between 50-100 mm^3^ and harvested splenocytes 3 days later (3 day post ALT-803 administration/day10 post tumor implantation. Primary tumor volumes on day 10 post tumor challenge were ∼100 mm3 for both ALT-803 and PBS treatment groups. As in non-tumor bearing mice, administration of IL-15SA/IL-15RαSu-Fc to mice induced the greatest effect on NK cells (3.5 fold, *p* < 0.001), followed by a significant increase in CD8^+^ and CD4^+^ T-cells (Figure 6A and 6B). Tregs were not significantly increased. When total NK activity was analyzed using day 3 splenocytes as effectors and 4T1 tumor cells as targets, IL-15SA/IL-15RαSu-Fc dramatically increased total NK-mediated cytotoxicity (Figure 6C), (E:T/100:1, *p* <0.001). Similar results were observed using enriched NK cells from the spleens of IL-15SA/IL-15RαSu-Fc treated tumor-bearing mice. These studies indicate that IL-15SA/IL-15RαSu-Fc has the potential to induce a potent NK-mediated anti-tumor response due to its ability to enhance total NK function in tumor-bearing mice.

### IL-15SA/IL-15RαSu-Fc showed significant anti-metastatic activity, dependent on CD8^+^ T cells as well as NK cells, and prolonged survival in 4T1 breast tumor model

**Figure 7:**
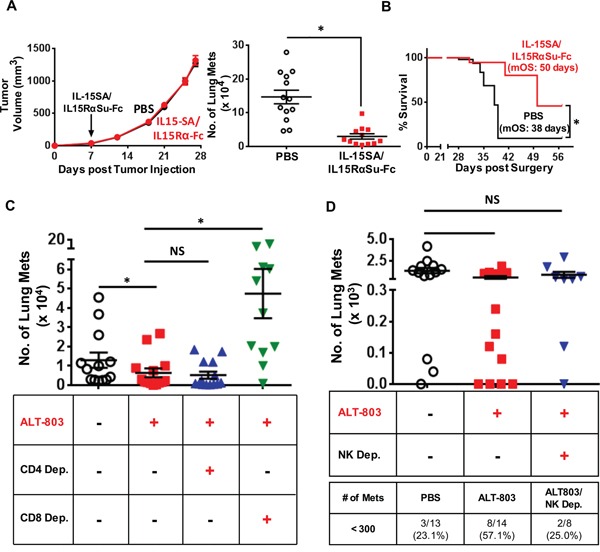
IL-15SA/IL-15RαSu-Fc shows significant anti-metastatic activity, dependent on CD8^+^ T and NK cells, and prolonged the survival of mice in the 4T1 breast tumor model **A.** Female Balb/c mice (n = 15/group) received 4T1 mammary tumor cells, followed by administration of IL-15SA/IL-15RαSu-Fc or PBS on day 7 post–tumor implantation. Primary tumor volume was measured once or twice per week, and the lungs were harvested and analyzed for pulmonary clonogenic cells when the tumor size reached ∼1200 mm^3^. *p < 0.05, statistical significance determined by the two-tailed Mann-Whitney *U* test. **B.** For the survival study, 4T1 tumor implantation and IL-15SA/IL15RαSu-Fc or PBS administration were performed as in (A). Primary tumors were excised on day 14 post 4T1 implantation, then the survival rates of IL-15SA/IL-15RαSu-Fc–treated (n = 6) and PBS-treated (n = 9) mice were examined. Median overall survival (mOS) for each group is shown. *p < 0.05, statistical significance determined by the Gehan-Breslow-Wilcoxon test. For **C.** T cell and **D.** NK cell depletions, 4T1 tumor implantation and IL-15SA/IL-15RαSu-Fc (ALT-803) or PBS administration were performed as in (A). Anti-CD8 (Clone 2.43) and anti-CD4 (GK 1.5) mAbs were used to deplete CD8^+^ and CD4^+^ T cells, respectively. Anti-asialo-GM1 Ab was used to deplete NK cells. Clonogenic metastatic analysis was performed as in (A). Also shown in (D) is the percentage of mice for each treatment group that had less than 300 metastases. Error bars represent SE of mean of number of clonogenic metastasis per group. *p < 0.05, statistical significance determined by the two-tailed Mann-Whitney *U* test. NS: no significance determined by the two-tailed Mann-Whitney *U* test.

To assess the functional consequences of IL-15SA/IL-15RαSu-Fc–mediated anti-tumor immunity and efficacy, we used the spontaneous metastasis model of 4T1 breast carcinoma, as the progressive invasion of 4T1 tumor cells to draining lymph nodes and other organs is similar to that seen in the advanced breast cancer in humans [24, 25]. We injected 1 μg of IL-15SA/IL-15RαSu-Fc on day 7 post–tumor implantation when the primary tumor size ranged between 50-100 mm^3^. IL-15SA/IL-15RαSu-Fc did not affect the primary tumor growth, but generated significant anti-metastatic activity, as the number of 4T1 metastases in the lung from IL-15SA/IL-15RαSu-Fc-treated mice decreased significantly (*p* < 0.001) (Figure 7A). Since primary tumors are surgically removed in patients with metastatic breast tumors, we modeled this scenario by performing surgical resection of the 4T1 primary tumor on day 14 and measured survival rates thereafter. Mice treated with IL-15SA/IL-15RαSu-Fc exhibited a significantly higher survival rate (*p =* 0.001), hence greater median overall survival (50 days) than that of the control group (38 days) (Figure 7B), which is in line with the high anti-metastatic property of IL-15SA/IL-15RαSu-Fc. Next, we wanted to determine which immune population(s) participated in IL-15SA/IL-15RαSu-Fc–induced anti-metastatic activity. CD4^+^/CD8^+^ T and NK cell depletions (Figure 7C and 7D) showed that the anti-metastatic property of IL-15SA/IL-15RαSu-Fc appeared to be most dependent on CD8^+^ T cells but not CD4^+^ T cells (Figure 7C). Focusing on NK cells, NK depletion abrogated the significant (*p =* 0.01) anti-tumor activity observed between non-treated and IL-15SA/IL-15RαSu-Fc–treated mice (Figure 7D). The role of NK cells was most evident in the proportion of mice that had less than 300 metastases (Figure 7D, untreated mice vs. NK depleted IL-15SA/IL-15RαSu-Fc–treated mice, *p =*0.09). These data are consistent with the published *in vivo* data pertaining to IL-15SA/IL15RαSu-Fc, which have shown that its mediation of anti-tumor and viral responses is dependent on CD8^+^ T and/or NK cells [16–18, 26]. Furthermore, these data correlate with the kinetic analysis of IL-15SA/IL-15RαSu-Fc–induced immune responses shown earlier, as NK and CD8^+^ T cell populations were most expanded by IL-15SA/IL-15RαSu-Fc administration (Figure 2).

### IL-15SA/IL-15RαSu-Fc therapy, particularly in conjunction with anti-CTLA-4 treatment, resulted in increased survival against experimental pulmonary metastasis of CT26 colon carcinoma cells

**Figure 8:**
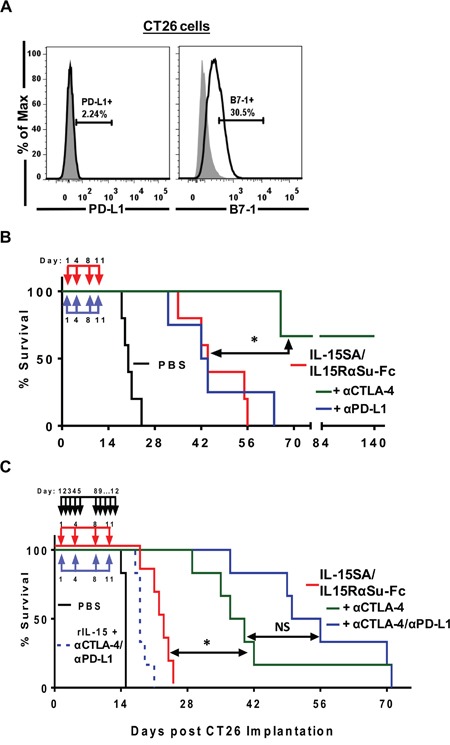
IL-15SA/IL-15RαSu-Fc prolongs the survival of mice against experimental pulmonary metastasis of CT26 colon carcinoma cells and in combination with checkpoint inhibitors, driven by anti-CTLA-4, resulted in synergistic enhancement of anti-tumor efficacy **A.** Surface expressions of PD-L1 and B7-1 (black lines) on CT26 cells were analyzed by flow cytometry. Isotype controls are shown in filled grey histograms. **B.** IL-15SA/IL-15RαSu-Fc (red down arrow) treated mice concurrently received either anti-CTLA-4 or anti-PD-L1 (blue up arrow), and their survival rates were measured. **C.** Mice received anti-CTLA-4 or in combination with anti-PD-L1 (blue up arrow) concurrently with IL-15SA/IL-15RαSu-Fc (red down arrow), and their survival was monitored. Also shown are mice that received rIL-15 (black down arrow) plus anti-CTLA-4/anti-PD-L1. *p < 0.05, statistical significance; NS: no significance determined by Gehan-Breslow-Wilcoxon test.

We tested the anti-tumor efficacy of IL-15SA/IL-15RαSu-Fc in an additional experimental metastasis tumor model using CT26 colon carcinoma cells. In a side-by-side survival study with rIL-15, we have shown that IL-15SA/IL-15RαSu-Fc generated a significantly higher anti-tumor activity than rIL-15 and prolonged survival against CT26 pulmonary metastasis [15]. In this study, we then examined the combination potential of IL-15SA/IL-15RαSu-Fc with the checkpoint inhibitors anti-CTLA-4 and anti-PD-L1. CT26 cells, similar to 4T1, have a very low level of PD-L1, but unlike 4T1, they highly express the CTLA-4 ligand, B7-1, on the surface [27–29]. To validate these findings, CT26 cells were stained with fluorochrome-labeled antibodies specific for PD-L1 and CTLA-4, followed by a flow cytometry analysis, which confirmed the poor expression of PD-L1 (2.24%) but significant expression of B7-1 (30.5%) on CT26 tumor cells (Figure 8A). In addition, we examined PD-L1 and B7-1 expressions on 4T1 and CT26 *in-vitro* and found that PD-L1, but not B7-1, expression was elevated in these cells after IFNγ treatment (Supplemental Figure 2). The results revealed that IL-15SA/IL-15RαSu-Fc when administered in combination with anti-CTLA-4, but not anti-PD-L1, synergistically increased survival of CT26-bearing mice (*p* < 0.01), (Figure 8B). Finally, we wanted to determine whether anti-PD-L1, albeit ineffective in combination with IL-15SA/IL-15RαSu-Fc (Figure 8B), provided survival benefit when incorporated into the combination treatment of IL-15SA/IL-15RαSu-Fc and anti-CTLA-4. The addition of anti-PD-L1 trended to improve the IL-15SA/IL-15RαSu-Fc plus anti-CTLA-4 treatment in the survival of CT26-bearing mice (Figure 8C). The same triple combination strategy, using rIL-15, was less effective than even IL-15SA/IL-15RαSu-Fc treatment alone (Figure 8C). Overall, administration of IL-15SA/IL-15RαSu-Fc exhibited a potent antimetastatic activity against not only 4T1 breast (Figure 7) but also CT26 colon carcinoma (Figure 8), and produced a synergistic anti-tumor response against CT26 pulmonary metastasis in combination with checkpoint inhibitors, driven mainly by anti-CTLA-4.

## DISCUSSION

To our knowledge, the results shown here report for the first time that IL-15SA/IL-15RαSu-Fc significantly promoted the development of “high effector” NK cells (Figure 4), which increased not only total function but also per-cell function of NK cells (Figure 5). In addition, IL-15SA/IL-15RαSu-Fc induced high levels of inflammatory cytokines, particularly IFN-γ (Figure 1), and promoted the development of innate memory CD8^+^ T cells expressing NKG2D (Figure 3); the latter immune response is a unique property of IL-15SA/IL-15RαSu-Fc that rIL-15 does not possess [14, 16]. We also demonstrate for the first time that IL-15SA/IL-15RαSu-Fc exhibited potent anti-metastatic activity, dependent on CD8^+^ T and NK cells, and prolonged the survival of mice, initially in the 4T1 breast tumor model, which resembles advanced breast cancer in humans (Figure 7). This anti-metastatic property of IL-15SA/IL-15RαSu-Fc was again exhibited in a second tumor metastasis model, involving CT26 colon carcinoma cells, with which we showed for the first time that the metastatic inhibition by IL-15SA/IL-15RαSu-Fc was more potent than that of rIL-15 (Figure 8A) and could be synergized with checkpoint inhibitors (Figure 8B and 8C). The studies reported here further define mechanistically the prior immune and anti-tumor studies of IL-15SA/IL-15RαSu-Fc [16–18].

Acute clinical toxicities have been a major concern for native rIL-15 administration due to highly elevated levels of pro- and anti-inflammatory cytokines such as IFN-γ, IL-6, TNF-α, and IL-10 [8]. In our murine model using a single intraperitoneal (i.p.) injection of 1 μg IL-15SA/IL-15RαSu-Fc, we also observed significant elevations of IFN-γ, TNF-α, and IL-10 (Figure 1A), but not IL-6 (Figure 1A; inset), which interestingly showed the greatest fold increase in the phase I clinical study of rhIL-15 [8] and was determined to be a primary cytokine mediator of toxicity in leukemia patients treated with chimeric antigen receptor effector cells [30, 31]. In our preclinical study, a single i.p. administration of 1 μg IL-15SA/IL-15RαSu-Fc significantly increased levels of inflammatory cytokines, but mice showed no observable toxicities at this dose (as determined by clinical observation and animal weights), possibly due to no significant change in serum IL-6 level (Figure 1A; inset). Our data reported here using Balb/c mice are consistent with data recently reported of an extensive non-clinical toxicity and pharmacodynamic studies of IL-15SA/IL-15RαSu-Fc in C57BL/6 mice and non-human primates [15]. Several phase I clinical studies have now been initiated with IL-15SA/IL-15RαSu-Fc that will also examine the levels of induced Th1 and Th2 cytokines.

Besides its increased potency and longer half-life compared with native IL-15 [11], IL-15SA/IL-15RαSu-Fc was shown to induce innate memory CD8^+^ T cells capable of non-antigen–specific killing in murine myeloma models [14, 16]. Our results are consistent with the published findings [14, 16], as we also found that IL-15SA/IL-15RαSu-Fc significantly increased IL-15 memory responders (CD122^+^ CD44^+^) in the CD8^+^ T cell compartment, in particular those having the innate (NKG2D^+^) phenotype (Figure 3). Notably, the kinetic profile of innate IL-15 memory responders in the CD8^+^ T cell population was very similar to that of NK cells (Figures 2 and 3), confirming the notion that IL-15SA/IL-15RαSu-Fc prevalently activates the innate arm of the immune response.

The subsets of CD8^+^ T cells such as IL-15 memory responders have been extensively described phenotypically and functionally [32–37]. However, significantly less analysis has been carried out on subpopulations of NK cells, which, unlike T cells, are classically considered non-antigen-specific and have no well-defined memory development [38, 39]. Because murine NK cells when exposed to IL-15SA/IL-15RαSu-Fc *in vivo* responded the highest among major immune cells in terms of total number and fold increase (Figure 2), it became a prerequisite to analyze the subsets of NK cells, particularly on day 3 post–cytokine complex treatment. We observed that IL-15SA/IL-15RαSu-Fc significantly promoted the development of “high effector” (CD11b^+^ CD27^hi^) NK cells, peaking on day 3 post cytokine complex treatment. It is interesting to note that the level of high effector NK cells returned to the baseline by day 7, whereas the significant level of “terminal effector” (CD11b^+^ CD27^lo^) NK cells still remained above the baseline (Figure 4A), indicative of the greater migratory capacity of high effector NK cells for tissue infiltration [21, 22].

Prior to the NK subset analysis mentioned above, we originally hypothesized that IL-15SA/IL-15RαSu-Fc would not have a significant impact on the per-cell function of NK cells, as nearly all NKs in naive Balb/c mice (day 0) showed an activated phenotype (∼80% of CD49b^+^ NKs were CD122^+^NKp46^+^) and maintained similar levels of the activated state throughout the *in vivo* kinetics study of immune cells exposed to IL-15-SA/IL-15Rα (Figure 4). As expected, IL-15SA/IL-15RαSu-Fc increased total splenic NK activity (Figure 5A). Unexpectedly, the significant increase in per-cell function of NK cells was observed (Figure 5B). We found that the effect of ALT-803 on NK and CD8+ T cells in tumor-bearing mice (Figure 6) was very similar to those in tumor-free mice (Figure 2). Regulatory T cells and monocytes/monocytic-MDSCs in tumor bearing mice also showed similar results as in tumor-free mice. As seen in tumor-free mice, ALT-803 significantly increased the number of neutrophils/granulocytic MDSCs in tumor bearing mice (Figure 6).

We attribute this enhanced per-cell function to increased development of high effector NK cells induced by IL-15-SA/IL-15RαSu-Fc. This finding also underscores the need for in-depth phenotypic and functional analyses of NK cell subsets. Adding to the potential complexity of these subsets, a cytometry by time of flight (CyTOF) analysis of human NKs revealed that there were 30,000 unique NK cell subsets found in 22 healthy individuals [40]. It has been postulated that this enormous number of unique NK subsets can be categorized on the basis of their ability to protect against different types of pathogens and, perhaps, also tumors [40–42].

We used two highly metastatic tumor models to examine the anti-tumor efficacy of IL-15-SA/IL-15RαSu-Fc. First, in the 4T1 breast tumor model, IL-15SA/IL-15RαSu-Fc was effective in reducing lung metastases, dependent on CD8^+^ T as well as NK cells, and increased the median overall survival from 38 to 50 days post–surgical resection of the primary tumor (Figure 7). In our study, CD8^+^ T cells and NK cells played a prominent role in inhibiting pulmonary 4T1 metastasis (Figure 7C and 7D). Because IL-15SA/IL-15RαSu-Fc significantly induces the expansion of high effector NK cells (Figure 4), which have been shown to express a high level of CXCR3 [21, 22] and exhibit superior migratory capacity [21, 22], incorporating IL-15SA/IL-15RαSu-Fc into a vaccine-based immunotherapy may synergistically enhance priming and activation of tumor-antigen specific T cells, mediated by this subpopulation of NK cells.

Next, we employed the CT26 pulmonary metastasis model not only to determine the extent to which the anti-metastatic property of IL-15SA/IL-15RαSu-Fc could be applied to a different tumor type, but also to combine IL-15SA/IL-15RαSu-Fc with an immune checkpoint therapy using, for example, anti-CTLA-4 or anti-PD-L1 antibodies. The immune checkpoint receptor ligands B7-1 and PD-L1 are upregulated on certain tumors and have been shown to inhibit T cell function by contributing to the tumor's ability to evade the immune system [43]. Thus, checkpoint inhibitors such as anti-CTLA-4 and anti-PD-1 or anti-PD-L1 antibodies can enhance T cell responses by breaking peripheral tolerance and preventing immune exhaustion [43, 44]. Additional combination therapies with immunostimulatory agents such as IL-15SA/IL-15RαSu-Fc, which promotes T cell proliferation and enhances their cytotoxicity, can potentially provide synergistic anti-tumor responses. In contrast to 4T1 tumor cells, which did not markedly express B7-1 and PD-L1, CT26 tumor cells expressed a low level of PD-L1 but a high level of B7-1 (see Results); hence their practicability for immune checkpoint combination therapy, specifically with anti-CTLA-4. Administration of IL-15SA/IL-15RαSu-Fc and anti-CTLA-4, but not anti-PD-L1, generated a synergistic anti-tumor response in CT26-bearing mice (Figure 8B), which is consistent with the observation that the efficacy of checkpoint inhibitors, in particular anti-PD-1 and PD-L1, is dependent on the surface expression of inhibitory ligands on tumor cells [45–47]. More interestingly, anti-PD-L1, albeit ineffective in combination with IL-15SA/IL-15RαSu-Fc in this tumor model, appeared to further improve survival when administered in conjunction with both IL-15SA/IL-15RαSu-Fc and anti-CTLA-4 (Figure 8C). We believe that CTLA-4/B7-1 blockade via anti-CTLA-4 induced IFN-γ secretion not only from T cells, but also from NK cells, which have been shown to express CTLA-4 in mediating IFN-γ production [48]. This likely resulted in increased expression of PD-L1 on CT26 tumor cells, rendering anti-PD-L1 efficacious. Our studies combining the IL-15 reagent with anti-CTLA-4 and/or PDL1 reflect our vision that the most effective therapies will be in combination with the emerging checkpoint inhibitors. We feel that increased survival in the CT26 tumor model (and control of macroscopic disease in the 4T1 model) can be further optimized by timing and sequencing of these reagents.

In conclusion, the results shown here support the use of IL-15SA/IL-15RαSu-Fc for anti-metastatic treatment against common non-hematologic tumors such as breast and colon carcinomas. Because IL-15SA/IL-15RαSu-Fc predominantly activated the innate arm of the immune system (Figures 2–4), a combination strategy involving the reagents that stimulate the adaptive arm of anti-tumor immunity, in particular recombinant vaccines or checkpoint inhibitors demonstrated in this study, may prove to be highly effective in the treatment of metastatic cancers.

## MATERIALS AND METHODS

### Animals

Female Balb/c mice were housed and maintained in microisolator cages under specific pathogen-free conditions and in accordance with the Association for Assessment and Accreditation of Laboratory Animal Care guidelines. All experimental studies were carried out under the approval of the National Institutes of Health (NIH) Intramural Animal Care and Use Committee.

### Tumor cells

4T1 murine mammary and CT26 murine colon carcinoma cell lines were purchased from American Type Culture Collection and maintained in the recommended medium.

### IL-15SA/IL-15RαSu-Fc (ALT-803) reagent

IL-15SA/IL-15RαSu-Fc (ALT-803) was generated as described previously [11] and kindly provided by Altor BioScience Corporation (Miramar, FL) under a Cooperative Research and Development Agreement (CRADA).

### Kinetic analysis of IL-15-SA/IL-15Rα-Fc–mediated immune responses

For each day, a group of naïve female Balb/c mice (n=3) was injected only once with IL-15SA/IL15-RαSu-Fc (1 μg in 100 μl, i.p.); their sera and spleens, including those from the control-PBS group (n = 3), were collected on day 7. Mouse sera were characterized for TH_1_ and TH_2_ cytokines using a cytokine multiplex analysis (Clinical Support Laboratory, NCI-Frederick). Mouse spleens were passed through a 70μM cell strainer and homogenized into a single cell suspension, followed by alkaline lysis of red blood cells, for antibody staining and flow analysis.

### Flow cytometry analysis; surface and intracellular marker assays

An LSR Fortessa (BD Biosciences, Franklin Lakes, NJ) was used for multiparametric flow cytometry analysis. A Live/Dead Fixable Blue Dead Cell Stain Kit (Molecular Probes; Thermo Fisher Scientific, Grand Island, NY) was used to exclude dead cells. The following murine monoclonal antibodies (mAbs) were used to stain Balb/c splenocytes to characterize immune cell subsets: FITC-CD8 (Clone 53-6.7; BD Biosciences), PE-CD49b (Clone DX5; BioLegend, San Diego, CA), PE-PD-L1 (Clone 10F.9G2; BioLegend), PerCP-Cy5.5-B7-1 (Clone 16-10A1; BioLegend), PerCP-Cy5.5-FOXP3 (Clone FJK-16s; eBioscience, San Diego, CA), PE-Cy7-CD122 (Clone Tm-b1; eBioscience), APC-NKG2D (Clone CX5; BioLegend), APC-Cy7-CD3 (Clone 17A2; BioLegend), Pacific Blue-CD44 (Clone IM7; BioLegend), AF700-CD4 (Clone GK1.5; eBioscience), BV605-CD19 (Clone 6D5; BioLegend), BV605-PD-1 (Clone 29F.1A12; BioLegend), BV510-NKp46 (Clone 29A1.4; BioLegend), Pacific Blue-CD27 (Clone LG.3A10; BioLegend), FITC-CD11b (Clone M1/70; BD Biosciences), AF700-CD11b (Clone M1/70; BioLegend), APC-Cy7-CD11c (Clone N418; BioLegend), BV510-CD3 (Clone 17A2; BioLegend), Pacific Blue-Ly6-G (Clone 1A8; BioLegend), BV605-Ly6-C (Clone HK1.4; BioLegend).

### NK cytotoxicity assay

Female Balb/c mice (n = 5–10/group) were given a single injection of IL-15SA/IL-15RαSu-Fc (1 μg in 100 μl, i.p.) or PBS (100 μl i.p.). On day 3, their spleens were harvested and processed as above. Mouse splenocytes were either used as effectors or purified for NK cells using the NK Cell Isolation Kit II (Miltenyi Biotec, San Diego, CA). YAC-1 target cells were labeled with ^111^In and co-cultured with bulk splenocytes or purified NK cells at 100:1, 10:1, and 1:1 effector-to-target ratios. For the NKG2D blocking assay, anti-mouse NKG2D (10 μg/ml; R&D Systems, Minneapolis, MN) or isotype control mAb was added to NK cells, incubated at room temperature for 20 min, then ^111^In-labeled YAC-1 cells were added. After incubation at 37° C for 4 hours, radioactivity in supernatant was measured using a γ counter (WIZARD^2^; PerkinElmer, Waltham, MA). Where indicated, NK activity was converted to lytic units (LU), as described by Wunderlich et al. [49].

### T/NK cell depletion study

Female Balb/c mice (n=15/group) were injected with 4T1 mammary tumor cells and given IL-15SA/IL-15RαSu-Fc (1 μg in 100 μl, i.p.) or PBS (100 μl i.p.) as above. T cell depletion was started on the day of tumor implantation using a daily dose (100 μg, i.p.) of anti-CD4 (Clone GK1.5) or anti-CD8 (Clone 2.43), purchased from BioXcell (West Lebanon, NH) for first the 3 days and once a week thereafter until harvest of the lungs (days 25–29). For NK depletion using anti-Asialo-GM1, it was reported that the greatest reduction of NK cell activity was seen on day 3 after one-shot injection, recovered on day 7, and rose to more than 50% of the original value on day 14 in nude and Balb/c mice [50]. Therefore, the following depletion schedule was used: briefly, 10 μl of anti-Asialo-GM1 (Wako Chemicals USA, Inc., Richmond, VA), diluted 1:5 in PBS, was injected 100 μl i.p. 1 day prior to IL-15SA/IL-15RαSu-Fc administration, then thereafter every 5-6 days until harvest of the lungs (days 25–30). The percent depletions of T and NK cells was checked on the day prior to IL-15SA/IL-15RαSu-Fc administration (CD4: 98.6%; CD8: 97.4%; NK: 91.6%) and 4 days after the last injection (CD4: 87.1%; CD8: 77.1%; NK: 33.7%).

### 4T1 anti-tumor and survival study

Female Balb/c mice (n = 15/group) were injected with 5 × 10^4^ 4T1 mammary tumor cells. Seven days after implantation, IL-15SA/IL-15RαSu-Fc (1 μg in 100 μl, i.p.) or PBS (100 μl i.p.) was administered. Tumors were measured twice a week. When the tumor volume (L × W^2^/2) reached 1000-1200 mm^3^, mice were euthanized, and their lungs were harvested and dispersed into single cell suspension, which was plated in the presence of 6-thioguanine (Sigma-Aldrich, St. Louis, MO). Twelve days later, cells were fixed with methanol, stained with 0.03% methylene blue, and the number of clonogenic metastatic cells was counted. For the survival study, primary tumors were surgically removed on day 14 post 4T1 implantation, then the survival of IL-15-SA/IL-15Rα-Fc-treated (N=6) or PBS-treated (N=9) mice was examined. Mice where surgery failed to control primary tumor (Dia. > 1.9 cm) after tumor resection were excluded for the survival analysis.

### CT26 anti-tumor and survival study

Female Balb/c mice (n = 6–8/group) were injected with 2x 10^5^ CT26 tumor cells intravenously (i.v.) on day 0. Treatment began 1 day later. In the rIL-15 treatment group, each mouse received 5 μg of rIL-15 (NCI, lot #: L0810009, Conc: 0.59 mg/ml, DOM: 12/03/08) i.p. daily, five times a week for 2 weeks. Along with rIL-15, animals also received both anti-mouse-PD-L1 antibody (clone 9G2; Biolegend) and anti-CTLA-4 antibody (clone UC10-4F10-11; Altor). In the IL-15SA/IL-15RαSu-Fc treatment group, each mouse received 4 μg of IL-15SA/IL-15RαSu-Fc i.v. twice a week. Along with IL-15-SA/IL-15RαSu-Fc, some mice receive either anti-mouse-PD-L1 antibody, anti-CTLA-4 antibody or both anti-mouse-PD-L1 and anti-CTLA-4 antibodies. The dose and schedule of these antibodies were 100 μg per injection administered twice a week for 2 weeks. Control mice received injections of PBS. Survival was set as an endpoint, and mice were monitored for weight changes thrice a week until the termination of the study.

### Statistical analysis

Unless specified, results of tests of significance are indicated as *p* values derived from a two-tailed Student's *t* test. All *p* values were derived at 95% using GraphPad Prism 6 statistical software for PCs (GraphPad Software, Inc., La Jolla, CA).

## SUPPLEMENTARY FIGURES



## Abbreviations

AICD: activation-induced cell death
conv.: conventional
CyTOF: cytometry by time of flight
IL: interleuken, IL-15SA/IL-15RαSu-Fc
ALT-803: IL-15 superagonist/IL-15RαSushi-Fc fusion complex
IFN-γ: interferon gamma
111In: indium 111
i.p.: intraperitoneal
i.v.: intravenously
LU: lytic units
MAb: monoclonal antibody
MDSC: myeloid derived suppressor cell
MHC: major histocompatibility complex
mOS: median overall survival
NCI: National Cancer Institute
NIH: National Institutes of Health
NK: natural killer
NS: no significance
PBS: phosphate buffered saline
rh: recombinant human
TCR: T cell receptor
TNFα: tumor necrosis factor alpha
Tregs: regulatory T cells
