# Supplementary material for: IL-15 superagonist/IL-15RαSushi-Fc fusion complex (IL-15SA/IL-15RαSu-Fc; ALT-803) markedly enhances specific subpopulations of NK and memory CD8+ T cells, and mediates potent anti-tumor activity against murine breast and colon carcinomas
Source: Oncotarget. 2016 Feb 18;7(13):16130–45. doi: 10.18632/oncotarget.7470 (PMC4941302; doi:10.18632/oncotarget.7470)
Supplement: Supplementary file 1 [file oncotarget-07-16130-s001.pdf]

SUPPLEMENTARY FIGURES

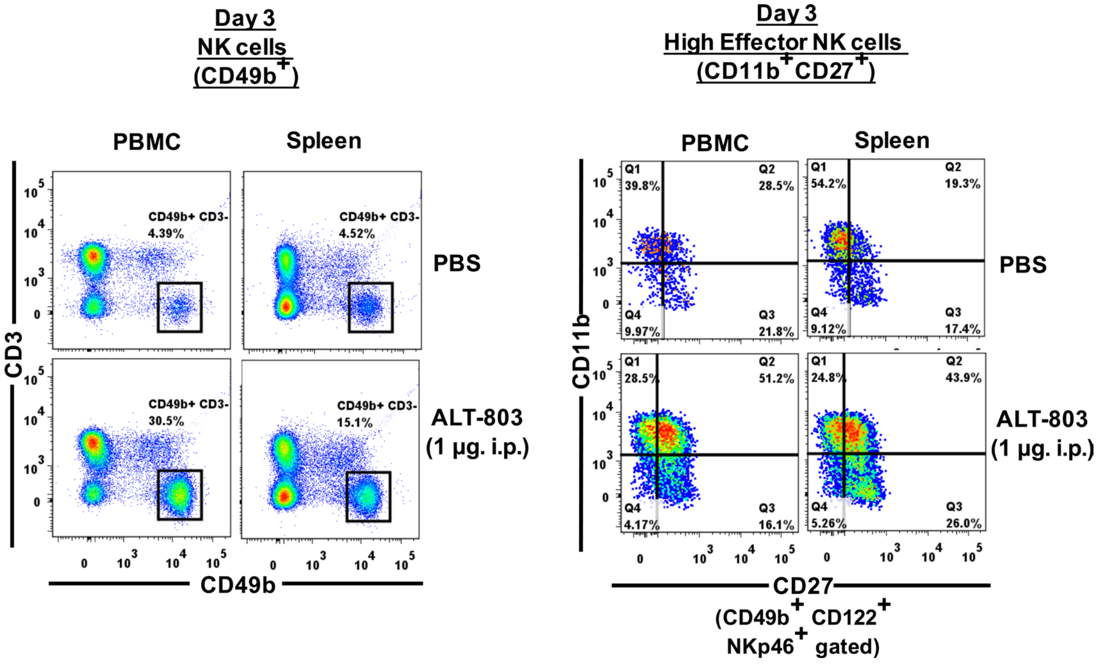

Supplemental Figure 1: IL-15SA/IL-15RαSu-Fc similarly enhances the expansion of NK cells in PBMCs in comparison with those in the spleens. A representative flow analysis of PBMC and splenic NK cells and their subsets from PBS and IL-15SA/IL-15RαSu-Fc-treated mice is shown.

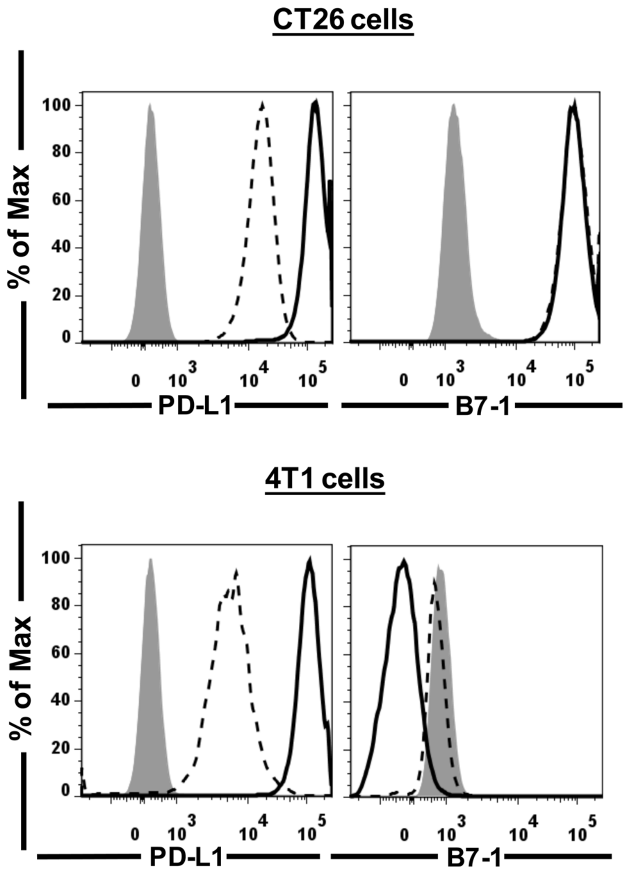

Supplemental Figure 2: IFN-γ increases PD-L1 but not B7-1 surface expression in CT26 and 4T1 tumor cells. Surface expressions of PD-L1 and B7-1 (IFN-γ treated (50 ng/ml for 24 hrs):solid lines; untreated: dashed lines) on CT26 and 4T1 tumor cells were analyzed by flow cytometry. Isotype controls are shown in filled grey histograms.
